# Transforming data to delight: AI-led campaign personalization shapes digital natives’ intention to accept over a TAM-VAM approach

**DOI:** 10.3389/frai.2026.1734151

**Published:** 2026-02-18

**Authors:** L. Durgha Devi, Thangaraja Arumugam

**Affiliations:** VIT Business School, Vellore Institute of Technology - Chennai Campus, Chennai, India

**Keywords:** AI acceptance, AI-led campaign personalization, digital natives, TAM, technology advantage, VAM

## Abstract

To explain consumers’ intention to accept artificial intelligence (AI)-led campaign personalization in cause-related marketing, this study integrates the technology acceptance model (TAM) and value-based adoption model (VAM). While TAM explains acceptance through functional and usability-driven evaluations of technology, it is insufficient to capture the emotional and value-driven judgments that are central to cause-related marketing contexts. VAM complements TAM, which is critical when AI-led campaign personalization is used to promote socially and ethically oriented initiatives. Focusing on digitally savvy consumers with high expectations for personalized initiatives, the study empirically examines these relationships using a purposive sample of 270 university students from Chennai, India. The data were collected through an online survey assessing key TAM factors, such as technology readiness, perceived usefulness, and perceived ease of use (PEOU), and VAM factors, such as perceived enjoyment and perceived value. Structural equation modeling using AMOS revealed that technology readiness strongly influences perceived ease of use (*β* = 0.48) and perceived enjoyment (*β* = 0.52), while perceived usefulness (*β* = 0.46) and perceived enjoyment (*β* = 0.49) significantly enhance perceived value. Perceived value emerged as the most substantial predictor of intention to accept AI-led campaign personalization (*β* = 0.60). These findings indicate that both functional and emotional benefits drive consumers’ intention to accept AI-led marketing. By integrating TAM and VAM, this study provides an empirical insight into how digital natives perceive and engage with AI-led strategies. The research offers theoretical contributions of AI intention to acceptance models and practical insights for businesses aiming to design personalized, value-driven, and emotionally engaging AI-led campaigns.

## 1 Introduction

The rise of artificial intelligence (AI) has transformed various industries, and brands are increasingly leveraging AI-led strategies to enhance consumer engagement (Gao and Liu, 2023). One of the most significant advancements in AI-led marketing is campaign personalization, which enables brands to deliver tailored messages and experiences to consumers. AI-led chatbots, a key component of this transformation, mimic human interactions to create personalized engagement, similar to their role in revolutionizing digital learning environments (Gujar, 2024). AI-led campaign personalization relies on natural language processing (NLP) and machine learning algorithms to understand consumer preferences and optimize brand interactions (Pérez et al., 2020). This approach enhances cause-related marketing initiatives by fostering deeper consumer-brand relationships and creating meaningful interactions between brands and consumers. Since the early developments of chatbot technology (Fryer et al., 2019), starting with ELIZA in 1966, AI-led engagement tools have evolved rapidly. Brands have integrated these tools into customer service, sales, and personalized marketing, significantly improving their ability to engage consumers (Shankar et al., 2022).

According to a recent market report—“Artificial Intelligence (AI) in Marketing – Global Strategic Business Report, 2025”—the global market for AI in marketing was estimated at USD$41.9 billion by 2030, growing at a rate of 26.7% between 2023 and 2030. This increase is driven by the increasing demand for automated, interactive brand experiences, just as personalized digital learning environments have gained momentum (Salnikova et al., 2022). The success of AI-led campaign personalization lies in its ability to provide real-time, tailored interactions that enhance consumer engagement. Just as chatbots in education offer students immediate feedback, problem-solving assistance, and personalized learning experiences, AI-led campaign personalization aims to create engaging, customized brand interactions that drive consumer interest (Pérez et al., 2020). By analyzing consumer behavior and preferences, AI-led campaign personalization can deliver content that resonates with digital native consumers, enhancing motivation and interest in cause-related marketing initiatives. Digital natives (Mertala et al., 2024), who have grown up with digital technology, expect highly personalized and interactive experiences from brands. AI-led campaign personalization meets these expectations by leveraging advanced data analytics to deliver relevant content, offers, and recommendations. Despite the growing acceptance of AI-led campaign personalization, understanding consumers’ perceptions and their intention to accept these interactions remains critical areas of study, as their engagement and acceptance directly influence the success of such campaigns. While previous studies (Tadimarri et al., 2024; Şenyapar, 2024; Singh and Ahmed, 2024) have examined AI’s impact on engagement, a majority of the research has primarily focused on companies’ perspective rather than consumers’ of AI-led strategies. Understanding how consumers perceive and have the intention to accept AI-led marketing efforts is essential for brands looking to maximize the effectiveness of their AI-led campaigns (Wilson et al., 2024).

AI-led campaign personalization is still in its early stages, and limited research has explored its intention to be accepted among digital native consumers, particularly within cause-related marketing initiatives. Cause-related marketing, which involves companies aligning with social or environmental causes, has gained significant traction as consumers increasingly prefer brands that demonstrate social responsibility (Skivko et al., 2021). AI-led campaign personalization has the potential to enhance cause-related marketing by delivering highly relevant, emotionally resonant messages that encourage consumer participation and support for causes. However, the extent to which consumers take AI-led cause-related marketing campaigns and the factors that influence their intention to accept require further exploration. This study explores the key factors shaping consumer intention to accept AI-led campaign personalization in cause-related marketing initiatives. Grounded in the technology acceptance model (TAM) and the value-based adoption model (VAM), the research examines how elements from both frameworks interact to influence consumers’ intention to accept. Specifically, it investigates the role of TAM factors, such as technology readiness, perceived ease of use (PEOU), and perceived usefulness, and VAM factors, such as perceived enjoyment and perceived value, in predicting consumers’ intention to accept AI-led campaign personalization in cause-related marketing. By bridging AI technology with cause-related marketing, this research aims to provide valuable insights for companies looking to optimize AI-led campaign personalization. Understanding the interplay between TAM and VAM factors will help brands develop AI-led marketing strategies that resonate with consumers and encourage positive engagement with a cause-related campaign.

## 2 Theoretical background

### 2.1 Technology acceptance model

The technology acceptance model (TAM) is a commonly cited framework for understanding individuals’ acceptance behavior toward technology (Choung et al., 2023). The fundamental factors that influence technology acceptance are perceived usefulness and perceived ease of use. Perceived usefulness refers to the belief that a technology enhances efficiency, making tasks more productive. In addition, perceived ease of use signifies the perception that the technology is straightforward, effortless to navigate, and does not require extensive effort to operate. These elements collectively determine whether individuals intend to accept or reject a particular technology. As proposed by Davis (1985), these factors are directly influenced by technology readiness, the third component of TAM. Technology readiness plays a crucial role in predicting individuals’ technology acceptance behavior. It represents an individual’s positive or negative perception of technology use (Stevens and Stetson, 2023).

### 2.2 Value-based adoption model

The technology acceptance model (TAM) is a widely used framework for assessing individuals’ acceptance of new technologies. However, its applicability is somewhat limited in predicting the decision-making process regarding technology adoption. To address this limitation, Kim et al. (2007) introduced the VAM, which builds upon TAM by incorporating the concept of perceived enjoyment and perceived value. VAM focuses on understanding the key motivations that drive consumers’ intention to accept and use a particular technology (Roostika, 2012). In the context of this study, perceived enjoyment and perceived value reflect consumers’ assessment of the trade-off associated with AI-led campaigns. When consumers view these campaigns as enhancing their overall experience and offering meaningful advantages, they are more likely to embrace and engage with them.

### 2.3 Integrated model of TAM and VAM in AI acceptance

This study combines the TAM and the VAM to offer a deeper insight into the factors influencing technology acceptance decisions. By incorporating both models, this approach captures how consumers evaluate the benefits before accepting and engaging with new technology.

**Figure 1:**
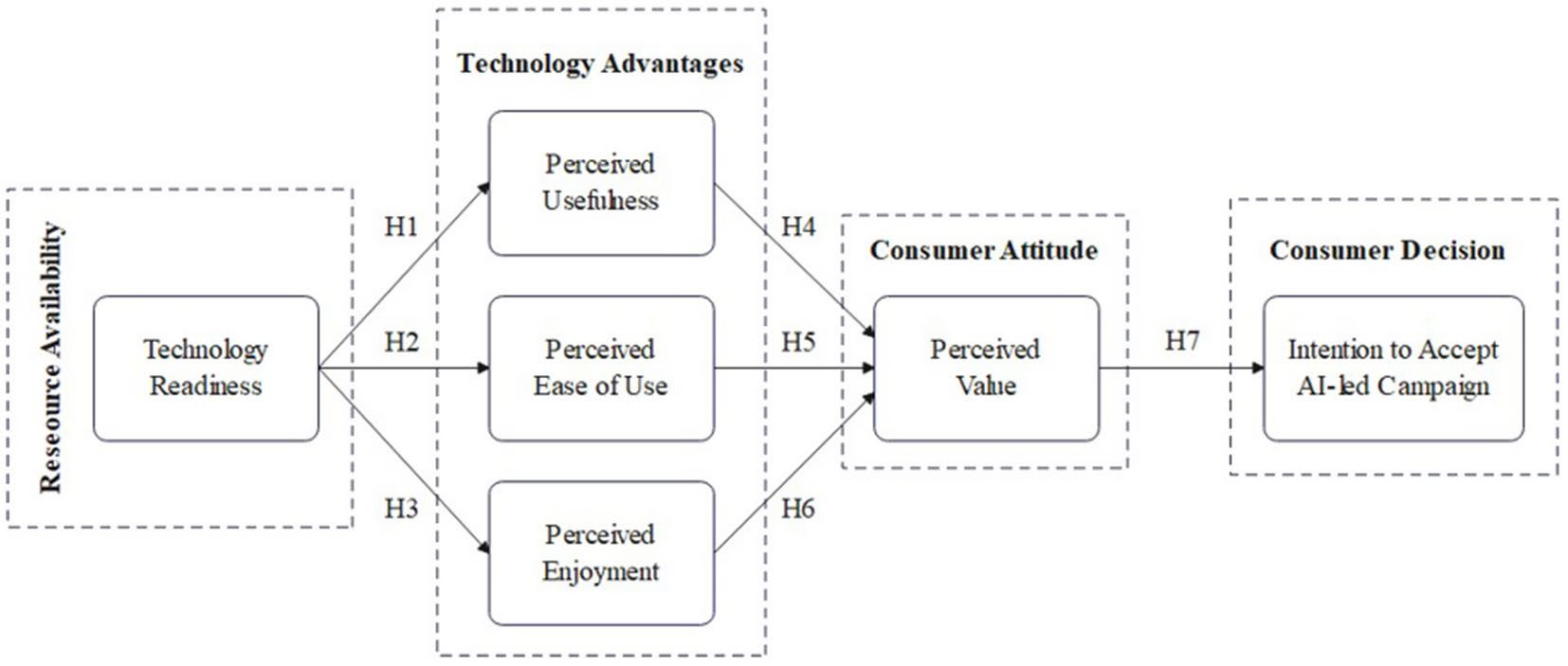
Proposed conceptual framework.

Prior studies on AI-led personalization have examined multiple technology acceptance models, such as the TAM, the Unified Theory of Acceptance and Use of Technology (UTAUT), the VAM, and the Theory of Planned Behavior (TAB). Among these models, Liao et al. (2022) identified VAM as the most effective model for predicting consumers’ acceptance of AI-led innovations. Recognizing the importance of individual values, numerous studies have integrated the VAM with other theoretical frameworks. However, within the realm of cause-related marketing, a research gap remains regarding consumers’ functional and usability-driven evaluations and emotional and value-driven judgments toward their intention to accept AI-led campaign personalization. Numerous studies have examined consumer acceptance through UTAUT and UTAUT2 models (Al-Adwan et al., 2023; Gansser and Reich, 2021; Venkatesh et al., 2012). While some research has applied the TAM, the majority have extended it with external variables to assess AI acceptance (Kelly et al., 2022; Gkikas and Theodoridis, 2021). Although research on AI-led marketing strategies is expanding, the VAM remains underexplored in this field. VAM highlights the importance of customizing cause-related content to align with consumers’ specific expectations, making it highly relevant to AI-led campaigns. Its core components—perceived enjoyment and overall perceived value—strongly correspond to the fundamental aspects of AI-led marketing initiatives. Given this alignment, integrating VAM with the TAM provides a strong framework for evaluating consumer intention to accept AI-led campaign personalization. This study advances existing literature by merging the TAM and VAM to assess consumer intention to accept AI-led campaign personalization, a perspective that has not been previously explored. Figure 1 shows the proposed conceptual framework for the study.

## 3 Literature review and hypothesis development

### 3.1 Relationship with the TAM

In the realm of AI-led marketing, TAM has been extensively applied to understand consumer perceptions of emerging technologies. Originally developed to explain consumer acceptance of information systems (Choung et al., 2023), the TAM remains a foundational framework for studying how individuals accept AI-led personalization. Just as the TAM has been extensively used to assess chatbot adoption in education, it provides valuable insights into consumer attitudes toward AI acceptance (Pérez et al., 2020).

Technology readiness refers to the availability and accessibility of modern technology that individuals or organizations can leverage to achieve their objectives. AI-led campaign personalization can utilize technology to enhance its existing systems and facilities once the required technology is available. Prior research has highlighted that, when sufficient technology is in place, consumers can complete tasks more conveniently, access resources seamlessly, and experience improved service. Moreover, technology readiness is closely linked to perceived usefulness, perceived ease of use, and perceived enjoyment. When systems are modernized, consumers often perceive them as more efficient and practical. Technological advancements can offer enhanced control, flexibility, and efficiency, helping consumers improve performance, streamline decision-making, and achieve better outcomes. Perceived usefulness in this context refers to the extent to which consumers believe AI-led campaign personalization enhances their overall brand experience and engagement. AI-led campaign personalization enables tailored content delivery, improving the effectiveness of cause-related marketing campaigns by fostering deeper emotional connections between brands and consumers. Research has consistently shown that perceived usefulness plays a pivotal role in influencing consumers’ behavioral intentions toward technology acceptance (Bhuiyan, 2024). Similarly, perceived ease of use describes how effortless and intuitive consumers find AI-led campaign personalization. In the same way that students’ acceptance of technology depends on their ease of use, consumer acceptance of AI-led marketing strategies is influenced by how seamlessly they interact with personalized content. Prior research (Bhuiyan, 2024; Teepapal, 2025; Tadimarri et al., 2024) suggests that when consumers perceive AI-led personalization as user-friendly and requiring minimal effort, they are more inclined to accept and engage with it. Similar to findings in chatbot technology acceptance research (Fryer et al., 2019; Roy Bhattacharjee et al., 2023; Salnikova et al., 2022), studies indicate that a favorable attitude toward AI-led marketing solutions leads to increased acceptance and engagement with personalized brand messaging. In AI-led campaign personalization, technology readiness enables consumers to customize systems and features to meet their specific needs, thereby enhancing their overall experience and performance after the integration of modern technologies. Building upon these insights, this study applies the TAM to examine the key factors influencing consumers’ intention to accept AI-led campaign personalization. The following hypotheses are proposed:

*H1:* Technology readiness positively influences the perceived usefulness of AI-led campaign personalization.

*H2:* Technology readiness positively influences the perceived ease of use of AI-led campaign personalization.

*H3:* Technology readiness positively influences the perceived enjoyment of AI-led campaign personalization.

### 3.2 Relationship with the VAM

The VAM explains consumer adoption of AI-led personalization in cause-related marketing by balancing perceived benefits against perceived sacrifices. In AI-led campaign personalization (Kim et al., 2019), consumers evaluate the trade-offs between the advantages of personalized engagement and concerns related to privacy, trust, and control over their data. Perceived benefits are a major determinant of consumer acceptance of AI-led marketing strategies. These benefits are categorized into perceived usefulness (utilitarian value) and perceived enjoyment (hedonic value) (Teo, 2011). Perceived usefulness in AI-led campaign personalization refers to the extent to which consumers believe that personalized marketing improves their brand experience. When consumers perceive that AI-led personalization offers more relevant product recommendations, better engagement, and enhanced purchase decision-making, they are more likely to see its value. Previous studies (Kim et al., 2007; Yu et al., 2017) confirm that perceived usefulness, mediated by perceived value, significantly influences technology acceptance. Perceived enjoyment refers to the emotional benefits that AI-led campaign personalization provides. Personalized marketing campaigns enhance engagement by making interactions with brands more dynamic, immersive, and tailored to individual preferences. Consumers who find AI-led campaign personalization enjoyable are more likely to engage with it, thereby strengthening their perceived value of AI-led marketing. Studies indicate that perceived enjoyment is a key predictor of perceived value (Mohamad et al., 2021; Suki and Suki, 2007).

In consumer behavior research, perceived value is the key factor driving the intention to accept. As defined by Zeithaml (1988), perceived value represents consumers’ overall perception of AI-led campaign personalization and its role in cause-related marketing initiatives. Value toward AI personalization is shaped by previous experiences, expectations, and trust in AI-led brand interactions. Within the VAM, perceived value serves as a mediator, linking perceived usefulness and perceived enjoyment to the acceptance of AI-led campaign personalization. If consumers recognize that AI-led campaign personalization offers more benefits than sacrifices, their likelihood of intention to accept increases. The following hypotheses are proposed:

*H4:* Perceived usefulness positively influences consumers’ perceived value of AI-led campaign personalization.

*H5:* Perceived ease of use positively influences consumers’ perceived value of AI-led campaign personalization.

*H6:* Perceived enjoyment positively influences consumers’ perceived value of AI-led campaign personalization.

*H7:* Consumers’ perceived value positively influences their intention to accept AI-led campaign personalization.

### 3.3 Integrating the TAM-VAM approach: AI-led personalization in cause-related marketing

The integration of the TAM, i.e., functional and usability-driven evaluations of technology, and the VAM, i.e., emotional and value-driven judgments, offers a structured approach to analyzing consumers’ intention to accept AI-led cause-related marketing initiatives (Babatunde et al., 2024; Malikireddy, 2024; Arango et al., 2023). While the TAM highlights the influence of technology readiness, perceived ease of use, and perceived usefulness, the VAM expands this understanding by incorporating perceived enjoyment and perceived value, shedding light on the benefit considerations that drive consumers’ intention to accept new technologies. By combining these models, businesses can better predict consumer engagement with AI-led marketing campaigns and optimize their strategies accordingly.

In AI-led campaign personalization, technology readiness, perceived usefulness, and perceived ease of use fall under functional benefits, and they are crucial in determining whether consumers will engage with automated marketing systems. When AI-led recommendations are intuitive, user-friendly, and seamlessly integrated into consumer interactions, individuals are more likely to perceive the technology as beneficial. According to Suki and Suki, (2007), the ease of navigating AI-generated marketing content enhances consumer perceptions of value, as it reduces cognitive effort and streamlines decision-making. Technology readiness in AI-led campaign personalization makes it more likely that consumers will perceive greater convenience and accessibility, ultimately increasing the rate of intention to accept (Liang et al., 2021).

Beyond functional benefits, AI-led marketing campaigns must also deliver emotionally engaging experiences to drive consumers’ intention to accept. Emotional benefits, particularly perceived enjoyment and perceived value, play a pivotal role. Perceived value toward AI-led marketing initiatives is enhanced when interactive and entertaining personalization techniques can make brand interactions more engaging. AI-led cause-related marketing initiatives that incorporate gamification, immersive storytelling, or personalized cause recommendations aligned with consumer values enhance both enjoyment and perceived value (Babatunde et al., 2024; Malikireddy, 2024). For example, AI-led marketing tools that allow consumers to visualize their impact on causes (e.g., demonstrating how their donations support a specific cause) can increase emotional involvement, making the campaign more persuasive. Consumers who enjoy these AI-led experiences are more likely to develop positive attitudes toward the brand and the technology, leading to higher engagement and long-term loyalty.

Despite the advantages of AI-led personalization, research suggests that consumer trust in AI systems is crucial. When consumers perceive that their data are being used transparently and ethically, they are more likely to engage with AI-led marketing campaigns. For instance, AI-led cause-related marketing initiatives that rely on extensive consumer data should clearly communicate their data protection policies to build trust (Teepapal, 2025; Gujar, 2024; Gao and Liu, 2023). When consumers recognize that AI-led cause-related marketing offers personalized, meaningful, and convenient engagement opportunities, they are more likely to develop a positive attitude toward the technology. Research on smart speakers (Gao and Huang, 2019), Internet of Things (IoT) adoption (Tsourela and Nerantzaki, 2020), and digital subscription (Sharma and Sijariya, 2024) services confirms that perceived value significantly influences consumers’ intention to accept. AI-led cause-related marketing campaigns can increase perceived value by demonstrating tangible benefits, such as more relevant cause recommendations, personalized donation options, and unified integration with social platforms. When consumers feel that AI-led marketing enhances their ability to support meaningful causes while improving their overall experience, they are more likely to accept and engage with the technology.

## 4 Research design

### 4.1 Samples and procedures

The study focuses on digital natives, a demographic known for their active engagement with technology and high expectations for personalized and interactive brand experiences (Lim et al., 2024). As future decision-makers, digital natives are particularly relevant for examining perceptions and intentions to accept AI-led campaign personalization in cause-related marketing initiatives. To explore this, an online survey was conducted among digital natives, specifically university students in Chennai, Tamil Nadu, India, using a purposive sampling technique. A total of 278 young adults participated in the survey, in which they were directed to a Google Form to assess their intention to accept AI-led campaign personalization in cause-related marketing through perceived effectiveness. To provide context for evaluation, participants were exposed to AI-led campaign personalization from leading brands, such as Cadbury’s “D for Dream”, Adidas’ “Run for the Ocean”, Coca-Cola’s “Recycle Me”, L’Oréal’s “AI Beauty for All”, Nike’s “AI-Powered Sneaker Recycle”, Nestlé’s “AI-Led Nutrition Scanner”, and Kinder Joy’s “AI-Led Storytelling”, among others. Each campaign was presented as a standardized set of visuals and descriptions, with all participants receiving the same stimuli to ensure consistency. The average exposure duration per campaign was approximately 2–3 min, allowing sufficient time to review and evaluate the campaign.

To ensure attentiveness and engagement, attention-check questions were included intermittently, verifying that participants were reading the content carefully. No explicit manipulations were applied, as the study aimed to assess participants’ natural perceptions and intention to accept AI-led personalization rather than experimentally altering campaign features. They were then asked to assess these campaigns based on their perceived effectiveness. Following data collection, 8 incomplete responses were removed, resulting in a final sample size of 270 participants.

### 4.2 Measures

The measurement of key factors in this study was conducted using established scales. Functional factors were assessed based on technology readiness, perceived usefulness, and perceived ease of use, comprising a total of 12 items, with 4 items per factor (e.g., “Using AI-led campaigns personalization helps me make informed procuring decisions about cause related initiatives” and “Interacting with AI-led campaigns personalization is straightforward and user-friendly”) adapted from Nuralam et al. (2024). Emotional factors were evaluated through perceived enjoyment and perceived value, using three items from three draws from Lai et al. (2024) (e.g., “AI-led campaign personalization captures my interest when promoting cause-related initiatives”). Perceived value was assessed using four items from Lai et al. (2024), including, e.g., “AI-led campaigns provide me with meaningful insights into cause-related initiatives in brands”. Finally, the intention to accept AI-led campaign personalization was measured using five items from Esiyok et al. (2024), with an example statement being, “I intend to engage with AI-led campaigns personalization when purchasing in brands with cause related initiatives now and in the future”. All items were rated on a 5-point Likert scale (1 = strongly disagree; 5 = strongly agree). The study used structural equation modeling (SEM) for data analysis, utilizing AMOS 24 software to conduct an in-depth examination of the relationships between key variables.

## 5 Empirical results and analysis

Table 1 reveals that, out of the 270 respondents, 54.81% are male and 45.18% are female, showing a fairly balanced sample with a slight male majority. The largest group of respondents is 18–20 years old (42.6%), followed by 21–23 years (25.9%). The majority of the respondents are undergraduates (61.5%), followed by postgraduates (27.8%) and school students (10.7%). A significant majority (78.51%) have seen AI-led campaign personalization by brands, while 21.48% have not. This indicates strong awareness among the respondents about AI-driven marketing. Approximately 72.59% of respondents said “Yes”, indicating that AI-led personalization motivates them to engage, and only 1.85% said “No”, while 25.55% said “Maybe”. This indicates a highly positive attitude toward AI-led personalization, with a large proportion of potential engagement, although some remain undecided. The sample mainly consists of young, educated undergraduates, most of whom are familiar with AI-led personalized campaigns by brands. Notably, a large majority feel motivated to engage with such campaigns, highlighting the effectiveness of AI-led personalization in capturing consumers’ intention to accept.

**Table 1:** Demographic response of the respondents.

Measure	Item	Frequency (N = 270)	Percentage (%)
Sex	Male	148	54.81
	Female	122	45.18
Age	<18 years	42	15.6
	18–20 years	115	42.6
	21–23 years	70	25.9
	24–26 years	43	15.9
Education	Schooling	29	10.7
	Undergraduates	166	61.5
	Postgraduates	75	27.8
Have you seen AI-led campaign personalization in brands?	Yes	212	78.51
	No	58	21.48
Does AI-led campaign personalization motivate you to engage with the campaign?	Yes	196	72.59
	No	5	1.85
	Maybe	69	25.55

### 5.1 Measurement model

The measurement model is presented in Table 2, with results demonstrating strong reliability and validity across all latent constructs. Factor loadings for all items range between 0.62 and 0.93, exceeding the minimum acceptable threshold of 0.60 (Hair et al., 2019), confirming good indicator reliability. Cronbach’s alpha values, ranging from 0.72 to 0.92, indicate strong internal consistency among the constructs. Similarly, composite reliability (CR) values between 0.71 and 0.90 further confirm the reliability of the measurement model, surpassing the recommended benchmark of 0.70. The average variance extracted (AVE) values for all constructs are above 0.50, ranging from 0.62 to 0.84, establishing satisfactory convergent validity. Overall, the measurement model ensures that the scales used for assessing technology readiness, perceived usefulness, perceived ease of use, perceived enjoyment, perceived value, and intention to accept AI-led campaign personalization are both reliable and valid for subsequent structural analysis.

**Table 2:** Construct reliability and validity.

Latent variable	Indicators	Factor loading	Cronbach alpha	Composite reliability	Average value extracted
Technology readiness	TR1	0.74	0.72	0.71	0.62
	TR2	0.70			
	TR3	0.81			
	TR4	0.68			
Perceived usefulness	PU1	0.66	0.76	0.73	0.76
	PU2	0.72			
	PU3	0.62			
	PU4	0.76			
Perceived ease of use	PEU1	0.82	0.84	0.80	0.64
	PEU2	0.77			
	PEU3	0.73			
	PEU4	0.69			
Perceived enjoyment	PE1	0.92	0.92	0.86	0.84
	PE2	0.67			
	PE3	0.83			
Perceived value	VL1	0.82	0.89	0.90	0.79
	VL2	0.79			
	VL3	0.67			
	VL4	0.90			
Intention to accept AI-led campaign personalization	AICA1	0.84	0.86	0.89	0.68
	AICA2	0.93			
	AICA3	0.76			
	AICA4	0.86			

The discriminant validity is provided in Table 3, with results confirming that all constructs are distinct, as the square root of AVE (TR = 0.74, PU = 0.84, PEOU = 0.80, PE = 0.92, PV = 0.78, ITA = 0.86) exceeds their inter-construct correlations. Technology readiness (TR) shows moderate associations with perceived usefulness (0.55) and strong associations with perceived value (0.61), suggesting that greater readiness enhances perceived value and usefulness. Perceived usefulness (PU) is strongly associated with perceived value (0.72) and enjoyment (0.56), indicating that usefulness drives value and enjoyment. Perceived ease of use (PEOU) correlates most with enjoyment (0.66) and intention to accept AI-led personalization (0.60), emphasizing the role of simplicity in fostering engagement and acceptance. Perceived value (PV) emerges as a central construct linking readiness, usefulness, and enjoyment. Finally, the intention to accept AI-led campaign personalization (ITA) is primarily driven by ease of use, enjoyment, and readiness, highlighting that consumers’ intention to accept depends on user-friendly, engaging, and relevant AI experiences.

**Table 3:** Discriminant validity–Fornell–Larcker criterion.

Construct	TR	PU	PEOU	PE	PV	ITA
TR	**0.78**					
PU	0.55	**0.87**				
PEOU	0.46	0.42	**0.80**			
PE	0.38	0.56	0.66	**0.91**		
PV	0.61	0.72	0.47	0.64	**0.88**	
ITA	0.50	0.36	0.60	0.54	0.49	**0.82**

Bold values indicated technology readiness, perceived usefulness, perceived ease of use, perceived enjoyment, perceived value and intention to accept AI-led campaign personalization.

### 5.2 Structural model

**Figure 2:**
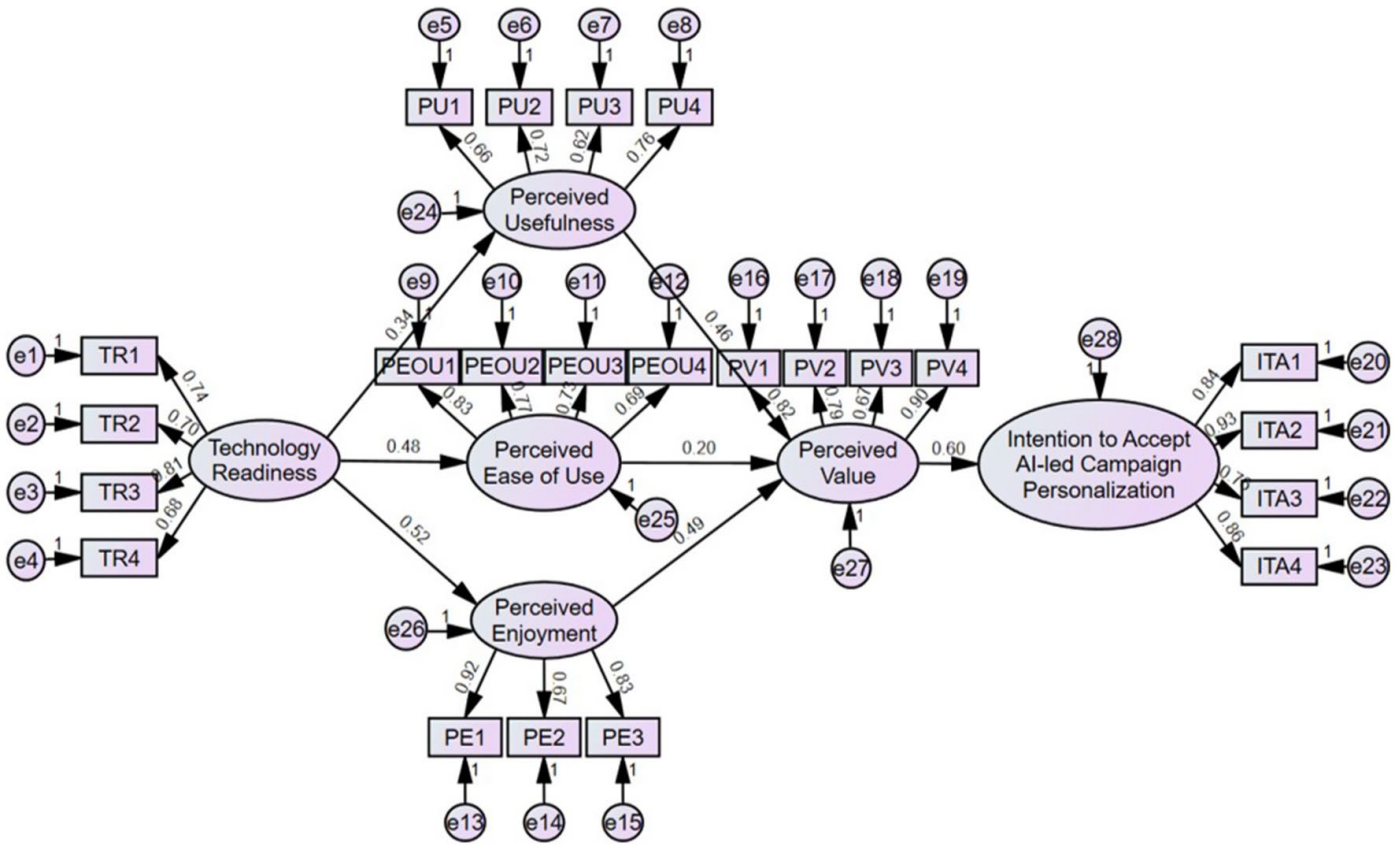
Structural equation model.

The structural model is presented in Table 4. It was analyzed using AMOS and reveals that all hypothesized relationships are positive and significant, with varying levels of effect strength. Figure 2 displays technology readiness (TR) and demonstrates a moderate influence on perceived usefulness (PU) (*β* = 0.34), indicating that higher readiness enhances the perception of AI’s utility. TR also exerts strong effects on perceived ease of use (PEOU) (*β* = 0.48) and perceived enjoyment (PE) (*β* = 0.52), suggesting that technologically confident individuals find AI-led systems easier and more enjoyable to use. Among the antecedents of perceived value (PV), perceived enjoyment (*β* = 0.49) and perceived usefulness (*β* = 0.46) show the strongest effects, while perceived ease of use (*β* = 0.20) exerts a relatively weaker influence. This highlights that emotional satisfaction and perceived utility contribute more to value perception than system simplicity. Finally, perceived value has the most substantial impact on consumers’ intention to accept AI-led campaign personalization (ITA) (*β* = 0.60), confirming its central role as a key mediator driving acceptance behavior. Overall, the AMOS-SEM results confirm a well-fitting structural model where technology readiness indirectly drives intention through usefulness, enjoyment, and perceived value, emphasizing the need for user-friendly, enjoyable, and value-driven AI marketing experiences.

**Table 4:** Path coefficient.

Hypothesized path	Standardized path coefficients (*β*)
H1 – Technology readiness –> Perceived usefulness	0.34
H2 – Technology readiness –> Perceived ease of use	0.48
H3 – Technology readiness –> Perceived enjoyment	0.52
H4 – Perceived usefulness –> Perceived value	0.46
H5 – Perceived ease of use –> Perceived value	0.20
H6 – Perceived enjoyment –> Perceived value	0.49
H7 – Perceived value –> Intention to accept AI-led campaign personalization	0.60

## 6 Research discussion and conclusion

### 6.1 Discussion and implication

As brands increasingly integrate AI-led campaign personalization into their marketing strategies, understanding consumer intention to accept is essential. This study uses the VAM, which emphasizes technology readiness and perceived value, as a comprehensive framework to examine consumers’ intention to accept. Specifically, the research investigates the viability of integrating the TAM and VAM to predict consumer acceptance of AI-led campaign personalization. This study builds upon existing literature by integrating VAM-related factors, perceived enjoyment, and perceived value with the fundamental constructs of the TAM, namely technology readiness, perceived ease of use, and perceived usefulness. To the best of our knowledge, this is the first study to explore the determinants of digital native students’ intention to adopt AI-led campaign personalization in Chennai, India. The findings contribute to the growing body of knowledge on AI-led personalization and offer practical implications for businesses, policymakers, educators, and AI technology designers. The insights derived provide strategic guidance for effectively implementing AI-led campaign personalization in branding efforts.

### 6.2 Conclusion

This study enhances the TAM by integrating essential elements from the VAM, such as perceived enjoyment and perceived value, to examine their collective influence on consumers’ intention to accept AI-led campaign personalization in brands. It is among the first studies to apply an integrated TAM–VAM framework to assess AI-led campaign personalization acceptance within cause-related marketing initiatives. By integrating TAM and VAM, this research offers valuable insights for AI technology developers, enabling them to refine their solutions to align with consumers’ internal and external needs, thereby improving intention to accept AI-led campaign personalization. The findings indicate that perceived value plays an equally significant role in influencing consumers’ intention to accept this technology. Additionally, technology readiness, perceived usefulness, and perceived enjoyment strongly shape consumers’ intention to accept AI-led campaign personalization. These results align with prior research by Kim et al. (2019), reinforcing the value of integrating TAM and VAM to assess consumer acceptance of AI-led personalization. From a practical perspective, these findings can guide digital marketers, AI developers, and brand strategists in designing and implementing AI-led campaign personalization strategies that effectively enhance consumer engagement and intention to accept.

### 6.3 Limitations and future research direction

The study employed a purposive sampling method, targeting digital native participants from colleges and universities who are familiar with AI-led campaign personalization in cause-related marketing initiatives. However, this approach limits the generalizability of the findings to the broader population of digital native consumers in Chennai. Future research should address this limitation by recruiting a more diverse sample, encompassing various consumer segments across different regions of India, to enhance representativeness. From a theoretical perspective, this study integrates key constructs from the original technology acceptance model (TAM), including technology readiness, perceived usefulness, and perceived ease of use, with constructs from the value-based adoption model (VAM), such as perceived enjoyment and perceived value. While this framework provides valuable insights, additional factors may further explain consumers’ intention to accept AI-led campaign personalization. Future studies could explore the role of consumer information processing in this context, particularly its mediating effect on AI acceptance. The study primarily focused on assessing value perception toward AI-led personalization by examining both functional aspects (technology readiness, perceived ease of use, and perceived usefulness) and emotional aspects (perceived enjoyment and perceived value). Furthermore, future research could explore how demographic variables such as age and gender moderate individuals’ perceptions and acceptance of AI technology. Despite these limitations, this study provides meaningful theoretical and practical contributions, offering valuable insights into consumers’ intention to accept the AI-led campaign personalization.

## Data Availability

The original contributions presented in the study are included in the article/supplementary material, further inquiries can be directed to the corresponding author.
